# Gate-Controlled Three-Terminal ZnO Nanoparticle Optoelectronic Synaptic Devices for In-Sensor Neuromorphic Memory Applications

**DOI:** 10.3390/nano15120908

**Published:** 2025-06-11

**Authors:** Dabin Jeon, Seung Hun Lee, Sung-Nam Lee

**Affiliations:** 1 Department of IT Semiconductor Convergence Engineering, Tech University of Korea, Siheung 15073, Republic of Korea; 2 Department of Semiconductor Engineering, Tech University of Korea, Siheung 15073, Republic of Korea

**Keywords:** ZnO, nanoparticle, 3-terminal, synapse, optoelectronic, neuromorphic

## Abstract

This study reports a gate-tunable three-terminal optoelectronic synaptic device based on an Al/ZnO nanoparticles (NPs)/SiO_2_/Si structure for neuromorphic in-sensor memory applications. The ZnO NP film, fabricated via spin coating, exhibited strong UV-induced excitatory post-synaptic current (EPSC) responses that were modulated by gate voltage through charge injection across the SiO_2_ dielectric rather than by conventional field effect. Optical stimulation enabled short-term synaptic plasticity, with paired-pulse facilitation (PPF) values reaching 185% at a gate voltage of −5.0 V and decreasing to 180% at +5.0 V, confirming gate-dependent modulation of synaptic weight. Repeated stimulation enhanced learning efficiency and memory retention, as demonstrated by reduced pulse numbers for relearning and slower EPSC decay. Wickelgren’s power law analysis further revealed a decrease in the forgetting rate under negative gate bias, indicating improved long-term memory characteristics. A 3 × 3 synaptic device array visualized visual memory formation through EPSC-based color mapping, with darker intensities and slower fading observed under −5.0 V bias. These results highlight the critical role of gate-voltage-induced charge injection through the SiO_2_ dielectric in controlling optical potentiation and electrical depression, establishing ZnO NP-based optoelectronic synaptic devices as promising platforms for energy-efficient, light-driven neuromorphic computing.

## 1. Introduction

Neuromorphic computing, which mimics the structure and function of the human brain, is gaining considerable attention as a promising paradigm for next-generation memory and processing technologies [1]. Central to these systems are artificial synaptic devices that emulate biological synaptic functions such as learning, memory retention, and signal transmission. In particular, optoelectronic synaptic devices, which respond to optical stimuli to emulate biological synaptic plasticity, have attracted growing attention due to their low-energy operation, fast response speed, and intrinsic compatibility with vision-based sensory systems [2]. Despite these advantages, optoelectronic synaptic devices also face notable challenges. Many conventional designs rely on two-terminal architectures, which inherently limit the precision of synaptic weight modulation and often suffer from trade-offs between optical sensitivity and electrical controllability [3]. To address these limitations, three-terminal device architectures have recently been explored to enable independent modulation of the channel conductivity via an external gate electrode [4]. This configuration decouples the electrical and optical pathways, allowing for more accurate control over learning, forgetting, and memory retention processes [5].

In terms of material selection, various semiconductors and hybrid systems have been investigated for optoelectronic synapse applications, including transition metal dichalcogenides (TMDs), perovskites, organic polymers, quantum dots, and metal oxides [2,3,4,5,6]. While materials such as MoS_2_ and hybrid perovskites exhibit high photoresponsivity, they often suffer from instability under prolonged illumination or ambient conditions [7]. In contrast, zinc oxide (ZnO) offers a unique combination of stability, processability, and functional optoelectronic properties, making it one of the most promising materials for light-driven neuromorphic systems [8,9]. ZnO possesses a wide bandgap (3.37 eV), high exciton binding energy, and strong UV absorption characteristics [10], enabling rapid generation of photocarriers and persistent photoconductivity (PPC)—a mechanism analogous to synaptic potentiation [11,12]. Its high chemical stability and compatibility with low-temperature, solution-based processes further enhance its appeal for large-area and flexible electronics. In particular, ZnO in the form of nanoparticles (NPs) exhibits an exceptionally high surface-to-volume ratio, which promotes enhanced light absorption, surface defect activity, and oxygen adsorption/desorption dynamics [13]. These surface phenomena are critical for modulating synaptic weight through EPSC and paired-pulse facilitation (PPF) mechanisms [14]. Moreover, ZnO NP-based devices exhibit strong field-effect sensitivity when configured in three-terminal architectures, allowing carrier injection-assisted modulation of charge density and synaptic behavior. Applying a gate voltage in this structure modifies the channel conductivity not purely via electrostatic field effect but by enabling limited carrier injection through the thin SiO_2_ dielectric from the p-type Si back gate. Under negative gate bias, electrons are partially injected into the ZnO NPs channel, increasing the free carrier concentration, prolonging persistent photoconductivity (PPC), and reducing the forgetting rate—key features for mimicking both short- and long-term synaptic plasticity observed in biological systems [9,15,16]. This charge injection mechanism works synergistically with photoexcitation, making ZnO NPs ideal for multifunctional optoelectronic synaptic systems. Overall, the integration of ZnO NPs into gate-tunable three-terminal optoelectronic synaptic devices provides a robust material–device platform for realizing in-sensor neuromorphic computing, enabling high-resolution memory storage, optical learning and biologically inspired plasticity within a scalable and energy-efficient framework [17].

## 2. Materials and Methods

A ZnO NPs thin film with a thickness of 50 nm was deposited on a thermally oxidized SiO_2_/Si substrate using a spin-coating method. The ZnO NPs dispersion ink (Sigma-Aldrich, Darmstadt, Germany) was prepared using isopropyl alcohol as the solvent. The spin-coating process was conducted at 2000 rpm for 60 s to ensure uniform film formation. Following deposition, the sample was soft-baked on a hot plate at 100 °C for 20 min to evaporate residual solvent and promote adhesion. Subsequently, Al electrodes with a thickness of 50 nm were thermally evaporated through a patterned shadow mask at a deposition rate of 2.5 Å/s to form the source and drain terminals. The final device consists of top Al electrodes and a ZnO NP channel deposited on a SiO_2_/p-Si substrate. The p-type Si (orientation: (100), n_h_= ~1.0 × 10^16^/cm^3^, resistivity: ~10 Ω·cm) serves as a bottom gate, isolated by a 100 nm thermally grown SiO_2_ layer. This three-terminal device enables optoelectronic synaptic behavior with gate-tunable modulation through a carrier injection-assisted mechanism.

Various characterization techniques were used to investigate the structural, optical and electrical properties of the fabricated ZnO NPs-based three-terminal optoelectronic synaptic device. The electrical performance of the device was characterized by measuring the current-voltage response under both dark and UV-illuminated conditions using a 365 nm light source. The surface morphology of the spin-coated ZnO NPs thin film was analyzed using atomic force microscopy (AFM, Nano Focus my-Scope plus, Seoul, Republic of Korea) and scanning electron microscopy (SEM, Coxem, Daejeon, Republic of Korea), while the crystalline structure was investigated through X-ray diffraction (XRD, Rigaku, Tokyo, Japan) and Raman spectroscopy (Andor Technology, Belfast, UK). Optical properties, including UV-visible absorption and photoluminescence (PL) measurements, were examined using a Xe lamp (Naku Technology, Hangzhou, China) and a 266 nm laser excitation source, respectively. The dark current and photocurrent characteristics of the ZnO NPs-based three-terminal device with an Al top to Al top electrode configuration were evaluated using a 365 nm UV light source. The photocurrent response was measured by illuminating the device with UV light while monitoring the drain current (I_DS_) at a fixed drain voltage. To investigate the gate-tunable photoresponse, different gate voltages (V_G_) ranging from −5.0 V to +5.0 V were applied to the bottom Si substrate. The influence of gate bias on carrier modulation was assessed by comparing current levels under dark and UV-illuminated conditions. To explore synaptic behavior, excitatory post-synaptic current (EPSC) responses were measured under a fixed drain bias of 1.0 V while applying varying optical stimuli such as changes in UV exposure time, light intensity, pulse frequency, and pulse number. Additionally, the influence of electrostatic gating was investigated by applying negative and positive gate voltages to the Si substrate, enabling control of both dark current and photocurrent levels.

## 3. Results and Discussion

### 3.1. Microstructural and Crystallographic Features of Spin-Coated ZnO NPs Thin Films

**Figure 1:**
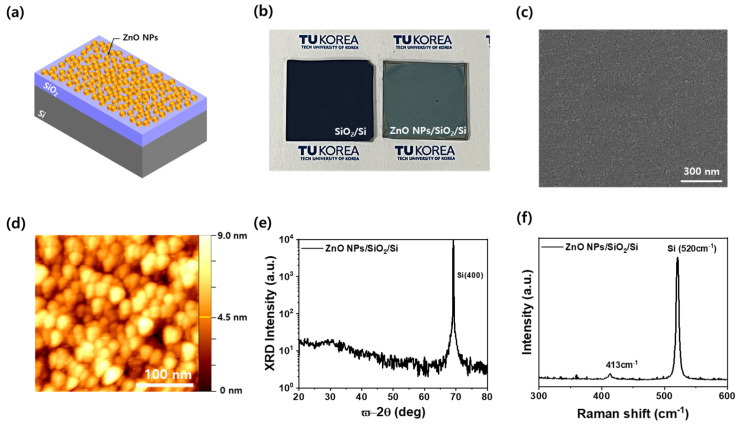
(**a**) Schematic illustration of the ZnO NP thin film deposited on SiO_2_/Si substrate (**b**) Photographs of the bare SiO_2_/Si substrate and the substrate coated with ZnO NP film, showing a noticeable color change after deposition. Surface morphologies of ZnO NPs films measured by (**c**) SEM and (**d**) AFM. (**e**) XRD ω/2θ scan and (**f**) Raman spectra of ZnO NPs films deposited on SiO_2_/Si substrate.

Figure 1a shows a schematic illustration of a ZnO nanoparticle (NP) thin film uniformly deposited on a SiO_2_/Si substrate. Figure 1b presents photographs comparing the bare SiO_2_/Si substrate and the ZnO NP-coated SiO_2_/Si sample. The bare substrate exhibits a deep bluish reflection due to the interference effect of the thermally grown SiO_2_ layer, whereas the ZnO NP-coated sample shows a lighter greenish hue. This color change arises from the surface modification caused by the ZnO nanoparticles, indicating successful deposition and uniform coverage. The observed contrast also suggests enhanced light scattering and absorption due to the presence of the ZnO NP film. Figure 1c shows a scanning electron microscopy (SEM) image of the ZnO NP film on a SiO_2_/Si substrate. The surface exhibits a densely packed and uniform distribution of ZnO nanoparticles without noticeable aggregation. The nanoparticles show a nearly spherical morphology, with sizes of ~30 nm. The absence of large voids or cracks indicates that the spin-coating process resulted in a continuous film. The SEM analysis further confirms that the solvent drying during spin coating facilitated a homogeneous nanoparticle assembly across the substrate. Figure 1d presents an atomic force microscopy (AFM) image of the ZnO NPs thin film deposited on a SiO_2_/Si substrate. The image reveals uniformly distributed NPs with a relatively consistent grain size, averaging approximately 28 nm, which closely matches the SEM results shown in Figure 1c. The measured root-mean-square (RMS) surface roughness is 3.54 nm, indicating a smooth and compact NPs layer. This uniform surface morphology suggests the effectiveness of the spin-coating process, where the centrifugal force and controlled solvent drying suppress NP aggregation and enable the formation of a densely packed film [18]. The rapid and uniform evaporation of the solvent during spin coating facilitates the self-organization of ZnO NPs, leading to a consistent nanoscale topography. The height variation across the scanned area remains within ~11 nm, further confirming the uniformity and flatness of the film. The crystalline of the ZnO NP thin film was examined by XRD ω-2θ scanning, as shown in Figure 1e. The spectrum reveals a dominant, sharp peak at approximately 69°, corresponding to the (400) diffraction of the underlying Si substrate. However, no distinct ZnO-related peaks, such as the (100), (002), or (101) reflections typically associated with hexagonal wurzite ZnO, are observed [19]. Instead, only a broad background signal is present, suggesting that the ZnO NPs exhibit random orientation and low crystallinity in the as-deposited state [20]. This is a common feature of spin-coated films that have not been thermally annealed. To further investigate the structural properties of the ZnO NPs [21], Raman spectroscopy is conducted, and the result is shown in Figure 1f. A clear peak appears around 413 cm^−1^, which corresponds to the E_2_ (high) vibrational mode of hexagonal wurtzite ZnO [22]. This mode is a signature of good local crystalline order within ZnO, even in nanocrystalline or disordered films. In addition, a strong peak at 520 cm^−1^ arises from the Si substrate. The presence of the E_2_ (high) peak confirms that the ZnO NPs maintain their intrinsic wurzite phase despite the absence of distinct diffraction features in the XRD pattern. Therefore, it is believed that the combined XRD and Raman analyses demonstrate that the film contains structurally identifiable ZnO NPs with short-range order, though lacking long-range crystalline coherence.

### 3.2. Optical Properties and Defect-Level Analysis of ZnO NPs Films

**Figure 2:**
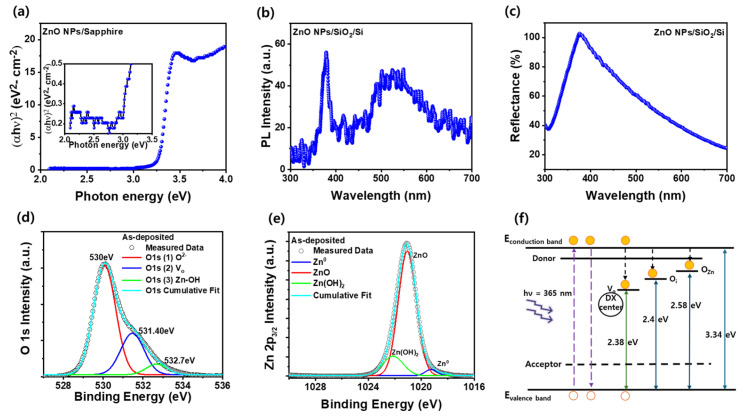
(**a**) UV–vis absorption spectrum of ZnO NPs on sapphire, with bandgap estimation and sub-bandgap absorption. The inset shows the absorption in the UV to visible range from 2.2 eV to 2.8 eV. (**b**) PL spectrum showing near-band-edge and deep-level emissions from ZnO NPs. (**c**) The reflectance spectrum of ZnO NPs on SiO_2_/Si shows strong UV absorption and high visible reflectance. (**d**) XPS O 1s spectrum indicating lattice oxygen, oxygen vacancies, and hydroxyl groups. (**e**) XPS Zn 2p_3/2_ spectrum showing Zn^2+^, Zn(OH)_2_, and minor Zn^0^. (**f**) Energy band diagram of ZnO NPs illustrating band-edge and defect-related states. Closed and open yellow circles represent electrons and holes, respectively. Dashed arrows (purple, black) show band-to-band and defect-related transitions; solid arrows (green, blue) indicate defect-level excitation and recombination.

To analyze the optical properties of the ZnO NPs/SiO_2_/Si film, UV-vis absorption, PL, and reflectance measurements were carried out, as shown in Figure 2a, b, and c, respectively. Figure 2a presents the UV–vis absorption spectrum of the ZnO NP film deposited on a sapphire substrate to eliminate interference from the SiO_2_/Si substrate and enable accurate determination of the optical absorption edge. A sharp increase in absorption is observed near 370 nm, which corresponds to the fundamental absorption edge of ZnO, indicating strong interband transitions. This sharp onset is characteristic of direct bandgap semiconductors and is commonly used to extract optical bandgap energy. As shown in the inset of Figure 2a, the absorption in the UV to visible range slightly increases from 2.2 eV to 2.8 eV, which may be attributed to optical transitions involving deep-level defect states, such as DX centers introduced by structural imperfections. The Tauc plot derived from the absorption spectrum yields an optical bandgap of approximately 3.34 eV, which corresponds to the intrinsic bandgap of ZnO NPs and confirms the presence of a well-preserved crystalline domain within the film. Figure 2b shows the PL spectrum of the ZnO NPs film deposited on SiO_2_/Si, exhibiting a strong near-band-edge (NBE) emission peak around 3.3 eV, which corresponds to the radiative recombination of free excitons near the ZnO band edge. In addition to the NBE peak, the spectrum exhibits pronounced deep-level emissions (DLEs) in the 2.0–2.8 eV, which are attributed to native point defects, including interstitial Zn atoms (Zn_i_) and oxygen vacancies (V_o_) [23]. These DLEs originate from a recombination process involving trapped charge carriers at defect states located within the bandgap. The presence of both sharp bandedge and broad defect-related emissions suggests that the ZnO NPs possess a partially crystalline structure along with a high density of intrinsic defects. Furthermore, the energy range of the DLEs closely matches the sub-bandgap absorption tail observed in the UV–vis spectra, reinforcing the interpretation that deep-level states contribute to the optical transitions in the material. Figure 2c presents the reflectance spectrum of the ZnO NPs film on a SiO_2_/Si substrate, measured over the 300–700 nm wavelength range. A significant dip in reflectance is observed between 370 and 380 nm, which corresponds to the intrinsic absorption edge of ZnO and is consistent with the absorption and PL results. This sharp drop indicates efficient absorption of UV light near the ZnO bandgap energy (~3.3 eV), confirming strong interband transitions. In contrast, the reflectance in the visible region (above ~400 nm) remains relatively high, implying that the film exhibits low absorption and high reflectivity in the visible range. This behavior indicates that the ZnO NP film functions as a UV-selective layer, strongly absorbing high-energy UV photons while allowing visible light to be reflected [24]. Figure 2d and Figure 2e present the XPS spectra of the ZnO NPs film deposited on a substrate, providing insight into the chemical states and defect structures of oxygen (O 1s) and zinc (Zn 2p_3/2_), respectively. These chemical states are closely correlated with the optical and electronic behavior of ZnO NPs films. These results help us understand the chemical states of zinc (Zn) and oxygen (O) in the material, which are closely related to its electronic properties and surface quality. In Figure 2d, the O 1s spectrum is deconvoluted into three peaks. The main peak at 530.0 eV corresponds to lattice oxygen (O^2−^) bonded in the Zn-O framework of ZnO. The second component, centered at 531.4 eV, is attributed to oxygen vacancies (V_o_)—sites where oxygen atoms are missing from the crystal lattice. These vacancies act as donor-like defect states within the bandgap and are known to influence carrier concentration and sub-bandgap optical transitions. The third peak, located at 532.7 eV, is assigned to surface hydroxyl groups (Zn–OH), which may form due to exposure to ambient moisture and can also contribute to shallow surface states [25]. Figure 2e shows the XPS Zn 2p_3/2_ spectrum for the ZnO NP film. The dominant peak located at 1022.4 eV is attributed to Zn^2+^ species (ZnO), confirming that Zn exists primarily in its oxidized state within the wurzite ZnO lattice. In addition to the main ZnO component, the deconvoluted spectrum reveals minor peaks corresponding to Zn(OH)_2_ (∼1022.1 eV) and metallic Zn^0^ (∼1019.2 eV). The presence of Zn(OH)_2_ is likely due to hydroxylation of the surface upon exposure to ambient moisture, while the trace amount of Zn^0^ suggests limited reduction or residual metallic zinc, possibly originating from incomplete oxidation or post-deposition reactions [26]. These XPS results indicate that the ZnO NPs are predominantly composed of Zn^2+^ species, with a small fraction of surface-related chemical states. The detection of Zn(OH)_2_ and Zn^0^ also implies the existence of defect-prone or chemically active regions on the nanoparticle surfaces. Together with the O 1s spectrum, which shows oxygen vacancies, these findings support the presence of defect states such as interstitial Zn and oxygen vacancies. These defects correlate well with the sub-bandgap absorption and deep-level emissions observed in the UV–vis and PL spectra, reinforcing their role as optically and electronically active states within the bandgap of ZnO NPs. To further illustrate these optical transitions, Figure 2f shows the energy band diagram of ZnO nanoparticles, including defect levels such as oxygen vacancies (V_o_) and interstitial zinc (Znᵢ) within the bandgap. These defects introduce deep-level states that correspond to the sub-bandgap absorption (2.2–2.8 eV) and broad PL emissions (2.0–2.8 eV) observed in Figure 2a,b. The deep-level states, including DX-like centers, can trap carriers and facilitate radiative recombination, explaining the presence of both near-band-edge and defect-related emissions. These results confirm that native point defects play a key role in determining the optical properties of ZnO NPs.

### 3.3. Photocurrent Behavior and PPC Mechanism in ZnO Nanoparticle Devices

**Figure 3:**
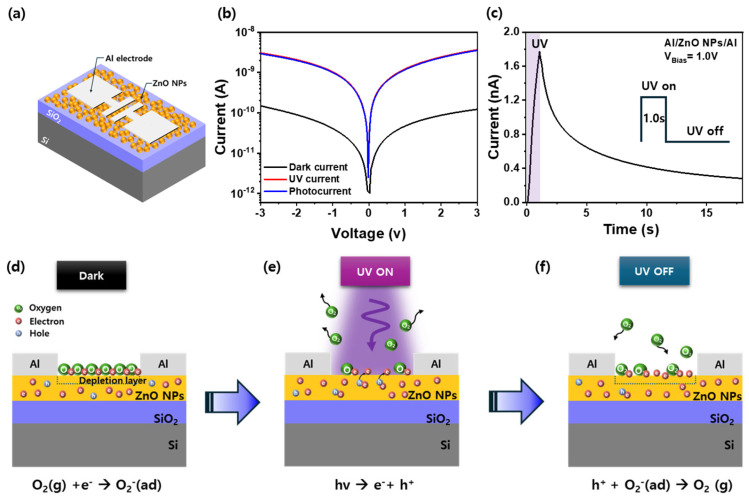
(**a**) Schematic of the Al/ZnO NPs/SiO_2_/Si optoelectronic synaptic device with a 2.0 mm × 8.0 mm ZnO NPs active layer. (**b**) I–V curves under dark and UV illumination, showing enhanced photocurrent under UV. (**c**) Time-dependent photocurrent response demonstrating persistent photoconductivity (PPC). (**d**–**f**) Photoresponse mechanism: (**d**) electron trapping by adsorbed O_2_ in the dark, (**e**) oxygen desorption and conductivity increase under UV, and (**f**) re-adsorption of O_2_ after UV off, leading to reduced conductivity.

Figure 3a presents a schematic illustration of the three-terminal Al/ZnO NPs/SiO_2_/Si optoelectronic synaptic device, where a ZnO NP thin film is deposited on a thermally oxidized Si substrate and patterned with Al electrodes. This configuration is designed to explore light-driven synaptic behavior based on photoconductive effects. Figure 3b shows the current (I)-voltage (V) curves of the device measured under dark conditions and UV illumination (λ = 365 nm). Under dark conditions at 3.0 V, the device exhibits a very low current of approximately 1.2 × 10^−10^ A, indicating excellent insulation and minimal carrier conduction. Upon UV exposure, the current significantly increases to 3.63 nA, demonstrating a pronounced photoconductive response. The resulting photocurrent—defined as the difference between the UV-illuminated and dark current—is approximately 3.63 nA, confirming the efficient photoresponsivity of the device. This enhancement is primarily attributed to the generation of electron-hole pairs upon UV excitation, which increases the concentration of free carriers in the ZnO NPs. In this process, shallow donor-like states such as oxygen vacancies play a supporting role by contributing to the background carrier population and promoting more efficient charge separation and transport. Figure 3c displays the transient photocurrent response of the device upon UV light illumination and subsequent removal, highlighting the dynamic photoresponse behavior. When the UV light is turned on, the photocurrent rapidly increases to 1.8 nA due to the immediate generation of photocurrent [27]. After UV light is turned off, the photocurrent does not return to the initial baseline but decays slowly over time, indicating the PPC effect. Unlike the initial current enhancement in Figure 3b, which is dominated by carrier generation, the slow decay in Figure 3c is primarily governed by the kinetics of oxygen re-adsorption and charge carrier trapping. As shown in Figure 2f, photogenerated carriers are temporarily trapped at deep-level defect states, such as the DX center associated with oxygen vacancies, which are located approximately 0.96 eV below the conduction band [28,29]. Simultaneously, oxygen molecules in the ambient atmosphere are gradually re-adsorbed onto the ZnO surface, capturing free electrons and reforming O_2_^−^ species. The mismatch in timescales between rapid oxygen desorption during illumination and slow re-adsorption in the dark leads to a delayed recovery of the depletion region and prolongs carrier lifetime, thereby sustaining the PPC response. Figure 3d–f further illustrates the underlying photoresponse mechanism based on surface charge modulation. In the dark state (Figure 3d), oxygen molecules (O_2_) from ambient air are adsorbed on the surface of ZnO NPs thin film, where they capture free electrons and form O_2_^−^ species, forming a depletion region that reduces conductivity [30]. Under UV illumination (Figure 3e), photogenerated holes (h^+^) neutralize the adsorbed O_2_^−^ ions, leading to oxygen desorption and a reduction in surface band bending [31]. This process narrows the depletion region and increases the density of free electrons, enhancing conductivity. After the UV light is turned off (Figure 3f), oxygen molecules are gradually re-adsorbed, trapping electrons to reform O_2_^−^ species. This process restores the depletion region and decreases conductivity. As illustrated in Figure 2f, the slow recovery of oxygen adsorption, in addition to carrier trapping at DX states, plays a key role in sustaining the PPC behavior by gradually depleting the free carriers.

### 3.4. Gate-Tunable Electrical and Photoresponsive Behavior of ZnO NP-Based Three-Terminal Devices

**Figure 4:**
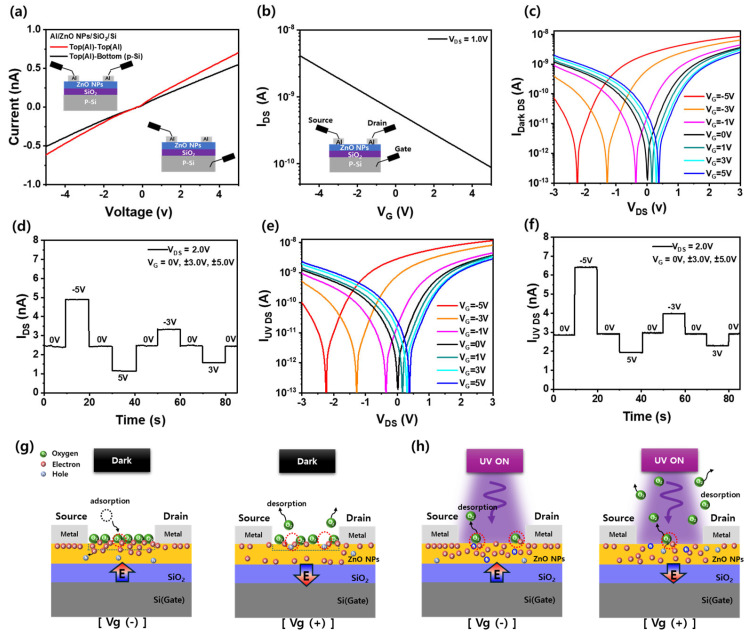
(**a**) I–V curves of Al–ZnO–Al (red) and Al–ZnO–SiO_2_–p-Si (black) configurations. The back-gate path shows nanoampere-level current, indicating gate leakage through SiO_2_ that contributes to channel modulation. (**b**) Log(I_DS_)–V_G_ characteristics of the Al/ZnO NPs/SiO_2_/Si three-terminal device measured under dark conditions at a fixed drain voltage of 1.0 V. (**c**) I_DS_–V_DS_ characteristics under various gate voltages in the dark. (**d**) Time-dependent I_DS_ response to sequential gate voltage steps in the dark. (**e**) I–V_DS_ characteristics under UV illumination at different gate voltages. (**f**) Time-dependent I_DS_ response to gate modulation under UV illumination. (**g**) Schematic illustration of carrier distribution and oxygen adsorption under negative and positive gate bias in dark conditions. (**h**) Schematic illustration of carrier transport and oxygen desorption under negative and positive gate bias during UV illumination. The ‘E’ in the arrow signal represents the movement of electrons in response to gate bias. The red dashed circle indicates the region where electron-bonded oxygen is, or will be, desorbed following reduction by holes.

Figure 4a presents the current-voltage (I–V) characteristics of the Al/ZnO NPs/SiO_2_/Si device measured between two different configurations: (i) between two top Al electrodes (red curve) and (ii) between the top Al electrode and the bottom p-type Si gate through the 100 nm SiO_2_ layer (black curve). The current level in both cases is in the nanoampere range, but importantly, a measurable gate current is observed in the Al–ZnO–SiO_2_–Si configuration. This result indicates that the SiO_2_ layer is not a perfect insulator under the applied voltage range (±5 V), and a slight leakage current flows from the Si back gate into the ZnO NP layer. Although thermally grown 100 nm SiO_2_ typically exhibits excellent insulating properties, the observed leakage current in our device is attributed to multiple factors related to processing and material characteristics. First, the ZnO NP film was deposited via spin coating and annealed at a relatively low temperature (100 °C), which may have left residual solvents or incomplete surface coverage near the ZnO/SiO_2_ interface. Such non-uniformities and potential pinholes can locally enhance electric field concentrations, resulting in increased leakage. Second, the porous and discontinuous morphology of the ZnO NP layer may facilitate localized current pathways, particularly when the top Al electrodes are deposited via shadow mask without lithographic isolation. This configuration can introduce edge leakage or metal penetration into surface pores. Finally, as shown in the I–V characteristics (Figure 4a) between the top electrode and back gate, the leakage current still remains in the low-nanoampere range under bias, which is sufficient for controlling synaptic behavior in a low-power regime without causing electrical breakdown. To address this issue in future work, we plan to introduce higher-temperature annealing, use atomic-layer-deposited ZnO or buffer layers to enhance dielectric integrity and implement patterned passivation to suppress leakage. Figure 4b shows the transfer characteristics of the Al/ZnO NPs/SiO_2_/Si three-terminal synaptic device plotted on a log(I_DS_)–V_G_ scale under dark conditions (V_DS_ = 1.0 V). The drain current increases nearly linearly with decreasing gate voltage, from +5 V to –5 V, without a distinct threshold or saturation region. This monotonic and symmetric log-linear behavior differs from conventional field-effect modulation and suggests that the observed current modulation originates from gate-induced carrier injection through the 100 nm SiO_2_ dielectric. Specifically, negative gate bias facilitates electron injection from the p-Si gate into the ZnO NP channel, increasing I_DS_, while positive bias drives electrons away, leading to current suppression. The absence of a sharp switching slope supports a leakage-assisted mechanism rather than an ideal electrostatic control. These results further confirm that the gate tunability in this device is dominated by SiO_2_ leakage currents, which are sufficient to induce synaptic-like behavior in low-conductivity ZnO nanoparticle networks. Figure 4c shows the output characteristics (I_DS_–V_DS_) of the Al/ZnO NPs/SiO_2_/Si three-terminal optoelectronic synaptic device measured in the dark under varying gate voltages from −5.0 V to +5.0 V. At V_DS_ = +1.0 V, the drain current increases with more negative gate bias, reaching 4.04 nA at V_G_ = −5.0 V, and significantly decreases to 75.8 pA at V_G_ = +5.0 V, demonstrating effective gate-dependent modulation of the channel current. Unlike conventional field-effect operation, this behavior is attributed to a weak but direct leakage current through the 100 nm-thick SiO_2_ layer, which modulates the carrier supply from the p-type Si gate to the ZnO NP channel. Negative gate bias induces electron injection toward the channel, increasing current, while positive bias suppresses carrier availability, leading to depletion and lower current, as shown in Figure 4g. Although this resembles a field-effect response, the relatively large leakage current through the oxide, verified by Figure 4a,b, indicates that direct current injection rather than electrostatic accumulation plays a dominant role. This leakage behavior is likely due to the combined effects of localized electric field enhancement across the relatively rough ZnO NP interface, the porous nature of the nanoparticle film leading to increased field penetration, and possible defect states or pinholes within the thermally grown SiO_2_ layer. This mechanism explains the distinct modulation characteristics compared to typical n-type ZnO thin film transistors. As shown in Figure 4c, when V_G_ = 0 V, the I_DS_–V_DS_ curve is almost symmetric and nearly linear around V_DS_ = 0 V, similar to the I–V behavior observed in Figure 4a for the Al(top)/ZnO NPs/Al(top) configuration. This implies that in the absence of gate voltage, the ZnO NP film maintains its intrinsic low-resistance behavior. When V_G_ is increased from 0 V to +5.0 V, the minimum current point shifts from V_DS_ = 0 V to +0.37 V, and the I_DS_ decreases at a fixed V_DS_, as observed in Figure 4b,c. Conversely, when V_G_ is decreased from 0 V to −5.0 V, the minimum current point shifts to −2.24 V and I_DS_ increases at V_DS_ = 1.0 V. This shift in the minimum current point reflects the gate-voltage-dependent injection or extraction of carriers through the slightly leaky SiO_2_ dielectric. Negative gate bias injects electrons into the channel, increasing its conductivity and displacing the conduction onset toward more negative V_DS_. In contrast, positive gate bias extracts electrons from the channel, reducing its conductivity and shifting the minimum current point toward more positive V_DS_. This behavior clearly deviates from that of a conventional field-effect device. As confirmed by the gate leakage current in Figure 4a, the observed modulation is better explained by carrier injection or removal through the slightly leaky SiO_2_ layer rather than by ideal field-induced accumulation. Particularly, as illustrated in Figure 4g, when a negative gate voltage is applied, electrons are injected from the p-Si gate into the ZnO NP channel through the SiO_2_ layer, increasing the carrier concentration and enhancing the I_DS_. On the other hand, a positive gate voltage pulls electrons from the ZnO NP channel toward the gate through the SiO_2_ layer, decreasing carrier concentration and resulting in a reduced I_DS_. A similar gate leakage-driven mechanism has been reported in a previous study on IGZO thin film transistors fabricated on SiO_2_/Si substrates [32], where devices with a large active area (36 mm^2^) exhibited not only significant drain current (I_DS_) but also large gate-to-drain (I_GD_) and gate-to-source (I_GS_) leakage currents. In contrast, devices with a small IGZO active layer (1.5 mm^2^) showed current flow only through the channel, with negligible leakage components. In our case, the abnormally shaped I_DS_–V_DS_ curves are likely influenced by the relatively large area (16 mm^2^) of the ZnO NP active layer, which increases the gate leakage effect and results in greater modulation of the channel current. This suggests that carrier injection through the gate dielectric can play a dominant role in current modulation, particularly in systems with porous or extended active layers. To further validate this mechanism, we plan to conduct systematic future studies using different active layer sizes, dielectric thicknesses, alternative oxide materials, and temperature-dependent measurements to quantify the carrier transport pathway and distinguish gate leakage effects from conventional electrostatic control. Figure 4d shows the time-resolved drain current (I_DS_) of the same device under dark conditions during a sequence of gate voltage steps (−5 V → 0 V → +5 V → 0 V → −3 V → 0 V → +3 V → 0 V) at V_DS_ = 2.0 V. Each gate transition results in reversible current changes due to carrier injection/removal through the leakage dielectric, confirming controllable current modulation based on direct gate-to channel conduction. Figure 4g schematically illustrates this behavior. When a negative gate voltage is applied, electrons are injected upward from the gate, populating the ZnO NP channel, reducing the depletion region and increasing conductivity. At positive gate bias, electrons in the ZnO NPs channel are pulled down or repelled, reducing carrier density and increasing the depletion width. In very low carrier-density ZnO NPs, oxygen molecules can also interact with these modulated carriers, reinforcing this dynamic. Figure 4e presents the I_DS_–V_DS_ output characteristics under 365 nm UV illumination, with gate voltages ranging from −5.0 V to +5.0 V. While a similar trend as the dark condition is observed, the overall current level increases significantly under UV illumination due to the generation of photocarriers. For example, at V_G_ = −5.0 V and V_DS_ = 1.0 V, the drain current increases from approximately 4.04 nA in the dark to 5.59 nA under UV illumination (Figure 4e), confirming that the UV excitation adds additional carriers to the leakage modulated base current. The I_DS_–V_G_ curves under both dark and UV conditions are presented in Figure S1. When V_DS_ is fixed at 1.0 V, the I_DS_ gradually decreases from 4.04 nA to 75.8 pA in the dark and from 5.59 nA to 86.6 pA under UV illumination, as V_G_ is swept from −5.0 V to +5.0 V. This modulation ratio of approximately 52 times in the dark and 64.5 times under UV demonstrates effective and tunable control of the synaptic signal through voltage-driven carrier injection, despite the absence of ideal field-effect transistor switching characteristics. 

Moreover, Figure 4d,f demonstrate the reliability of repeated measurements by showing consistent modulation of I_DS_ in response to a sequential gate voltage sweep (0 V → −5 V → 0 V → +5 V → 0 V → −3 V → 0 V → +3 V → 0 V) under both dark and UV conditions, respectively. The current increases and decreases appropriately with each gate voltage step, further verifying the gate-dependent and light-sensitive characteristics of the device. Photogenerated holes neutralize adsorbed O_2_^−^ species, promoting oxygen desorption and releasing trapped electrons into the channel. This combined effect of photocarrier generation and gate-induced electron modulation leads to a synergistic enhancement in conductivity, especially under negative gate bias. Figure 4f presents the time-dependent I_DS_ response under continuous UV illumination (λ = 365 nm, V_DS_ = 1.0 V) with gate voltage steps applied from 5.0 V to +5.0 V. The current increases to ~6.3 nA at −5 V due to the combined effect of UV-generated carriers and gate-driven electron injections. At 0 V and +5 V, the current drops to ~2.8 nA and ~2.0 nA, respectively, confirming that UV illumination enhances baseline conductivity across all gate voltages. The reversible and repeatable current modulation demonstrates reliable electrical tunability under light, further supporting the dual optical-electrical control of the device. Figure 4h illustrates the photoresponse mechanism under UV illumination, compared to the dark-state behavior shown in Figure 4g. Under negative gate bias and UV illumination, electrons are directly injected into the ZnO NP channel from the p-type Si back gate through the leaky SiO_2_ layer, while photogenerated holes facilitate oxygen desorption. This dual process increases carrier density and reduces the depletion region, leading to enhanced photocurrent. In contrast, positive gate bias pulls electrons away from the ZnO channel and toward the back gate, and although UV-generated holes still promote oxygen desorption, the diminished electron population in the channel limits conductivity, leading to a wider depletion region and lower current. Additionally, under positive gate bias, minority carriers (holes) of n-ZnO NPs are driven toward the ZnO surface, where they can extract electrons from absorbed O_2_^−^ species, facilitating oxygen desorption. However, due to insufficient replenishment of electrons through the back gate, the conductivity remains low. Nevertheless, because the concentration of photogenerated carriers under UV illumination is significantly higher than in the dark, the drain current in the UV condition remains greater than in the dark conditions at the same gate voltage. Thus, while UV illumination enhances conductivity, the gate bias modulates current primarily by directly controlling the electron supply via leakage paths, not by conventional field effect, and this governs the extent of surface oxygen interaction and the carrier dynamics of the device.

### 3.5. Short-Term Synaptic Plasticity in Al/ZnO NPs/SiO_2_/Si 3-Terminal Optoelectronic Synaptic Devices

**Figure 5:**
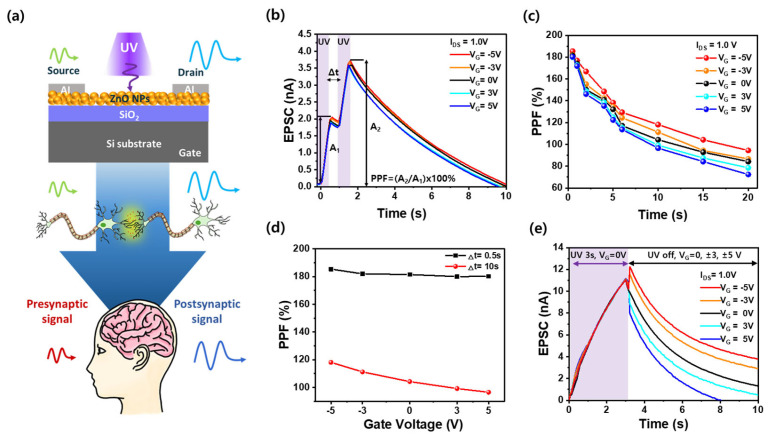
(**a**) Schematic illustration of the three-terminal Al/ZnO NPs/SiO_2_/Si optoelectronic synaptic device, mimicking a biological synapse by converting optical stimuli into electrical responses. (**b**) PPF characteristics of the Al/ZnO NPs/SiO_2_/Si synaptic device under various gate voltages. Two consecutive UV pulses (pulse width: 0.5 s) with an inter-pulse interval of 0.5 s were applied. (**c**) PPF as a function of different time intervals under a constant 0.5 s pulse exposure time for ZnO NP-based synaptic device. (**d**) PPF index measured at fixed delay times (Δt = 0.5 s and 10 s) as a function of gate voltage. (**e**) EPSC decay characteristics following a 3.0 s UV pulse (at V_G_ = 0 V) and subsequent application of various gate voltages.

Figure 5a shows a schematic illustration of the three-terminal optoelectronic synapse device based on an Al/ZnO NPs/SiO_2_/Si structure. Upon UV illumination and gate voltage modulation, the device generates an excitatory post-synaptic current (EPSC) between the source and drain terminals, mimicking the signal transmission in a biological synapse. This configuration emulates the transformation of a presynaptic optical stimulus into a post-synaptic electrical response, enabling the hardware implementation of synaptic plasticity. Figure 5b shows the results of paired-pulse facilitation (PPF) measurements using two consecutive UV pulses with an inter-pulse interval (Δt) of 0.5 s to assess short-term synaptic plasticity through changes in EPSCs [33]. The PPF was calculated as the ratio of the second EPSC (A_2_) to the first EPSC (A_1_), i.e., PPF = (A_2_/A_1_) × 100%. A higher PPF ratio indicates stronger synaptic facilitation. The device exhibited PPF behavior under different voltages ranging from −5.0 V to +5.0 V. The maximum PPF value reached 185% at V_G_ = −5.0 V and decreased slightly to 180% at +5.0 V, indicating that negative gate bias improves charge retention and short-term synaptic plasticity. Figure 5c shows the dependence of the PPF index on the inter-pulse interval (Δt) under various gate voltages. At V_G_ = −5.0 V, the PPF decreased from 185% to 94% as Δt increased from 0.5 s to 20 s. In contrast, at V_G_ = +5.0 V, the PPF dropped more steeply from 180% to 72.4%, indicating a faster decay of short-term memory. These results suggest that a negative gate bias enhances charge retention by modulating the surface potential and suppressing recombination, thereby preserving more photogenerated carriers within the ZnO NP channel. This leads to a stronger post-synaptic response and improved short-term synaptic plasticity compared to the positive bias condition. Figure 5d compares the PPF values at two fixed inter-pulse intervals (Δt = 0.5 s and 10 s) under various gate voltages. At Δt = 0.5 s, the PPF values remain relatively high and stable (185 ~ 180%) across all gate voltages, indicating robust short-term facilitation immediately after stimulation. In contrast, at Δt = 10 s, the PPF values decrease significantly with increasing gate voltage, from 118% at −5.0 V to 96% at +5.0 V. This trend demonstrates that short-term synaptic plasticity becomes more sensitive to gate control at longer delay times, where a more negative gate bias helps retain photogenerated carriers and sustain synaptic strength over time. Figure 5e shows EPSC decay characteristics measured after 3.0 s of UV illumination at V_G_ = 0 V, followed by switching to different gate voltages in the dark. Although all measurements began from the same initial photoconductive state, the relaxation behavior varied depending on the applied gate voltage. A more negative gate bias (e.g., −5.0 V) resulted in a slower decay and high residual EPSC, whereas a positive gate bias (e.g., +5.0 V) accelerated carrier recombination. These results confirm that the relaxation dynamics of photogenerated carriers, and thus short-term synaptic plasticity, can be effectively modulated by the gate voltage through electron injection or depletion via leakage through the SiO_2_ layer rather than conventional field-induced carrier control.

### 3.6. Enhanced Short-Term Synaptic Plasticity via Gate-Controlled Optical Stimulation

**Figure 6:**
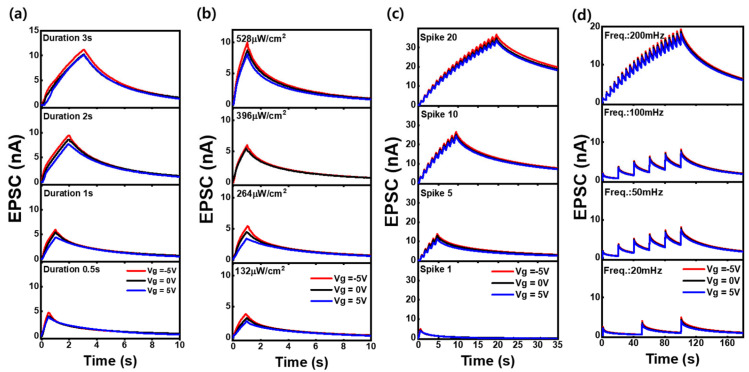
Time-dependent EPSC responses of the ZnO NPs-based three-terminal optoelectronic synaptic device under various optical stimulation conditions with gate voltages of −5.0 V and +0.5 V: (**a**) UV exposure time (0.5 to 3.0 s), (**b**) UV intensity (132 to 528 µW/cm^2^ at a fixed exposure duration of 1.0 s), (**c**) number of UV pulses increased from 1 to 20 with a pulse width of 0.5 s and 50% duty cycle, and (**d**) UV pulse frequency varied from 20 to 200 mHz at a fixed pulse width of 0.5 s.

Figure 6a–d presents the time-dependent EPSC responses of the ZnO NPs-based three-terminal optoelectronic synaptic devices under varying optical stimulation conditions: (a) UV exposure time (0.5 to 3.0 s), (b) light intensity (132 to 528 µW/cm^2^ at fixed 1.0 s exposure), (c) pulse number (1 to 20) with a pulse width of 0.5 s and 50% duty cycle, and (d) frequency (20 mHz to 200 mHz) at fixed pulse width of 0.5 s. All measurements were conducted under gate voltages of −5.0 V and +0.5 V. In all cases, the EPSC amplitude and decay behavior are strongly modulated by the gate voltage. Negative gate bias leads to larger EPSC peaks and slower decay, while positive gate bias results in weaker and faster-decaying EPSC responses. This gate-tunable behavior arises from the gate-induced electron injection or removal through leakage across the SiO_2_ dielectric, which modulates carrier concentration in the n-type ZnO NP channel. Under negative gate bias, electron accumulation enhances photoconductivity and facilitates charge retention, thereby prolonging the synaptic response. Conversely, positive gate bias depletes electrons from the channel, resulting in reduced photocurrent and shorter EPSC duration. Furthermore, increased UV exposure time, light intensity, pulse number, and stimulation frequency effectively enhance the synaptic memory characteristics by promoting charge trapping and accumulation. Notably, the application of negative gate bias consistently amplifies this enhancement, yielding higher EPSC levels and prolonged retention across all optical stimulation conditions. These results demonstrate short-term synaptic plasticity characteristics and suggest that negative gate voltage can effectively strengthen and prolong optical memory responses. Such controllability highlights the promise of ZnO NPs-based optoelectronic synaptic devices for implementing neuromorphic memory functions through gate-coupled carrier supply rather than transitional field-effect mechanisms.

### 3.7. Enhanced Learning and Memory Retention in ZnO NPs-Based 3-Terminal Optoelectronic Synaptic Devices

**Figure 7:**
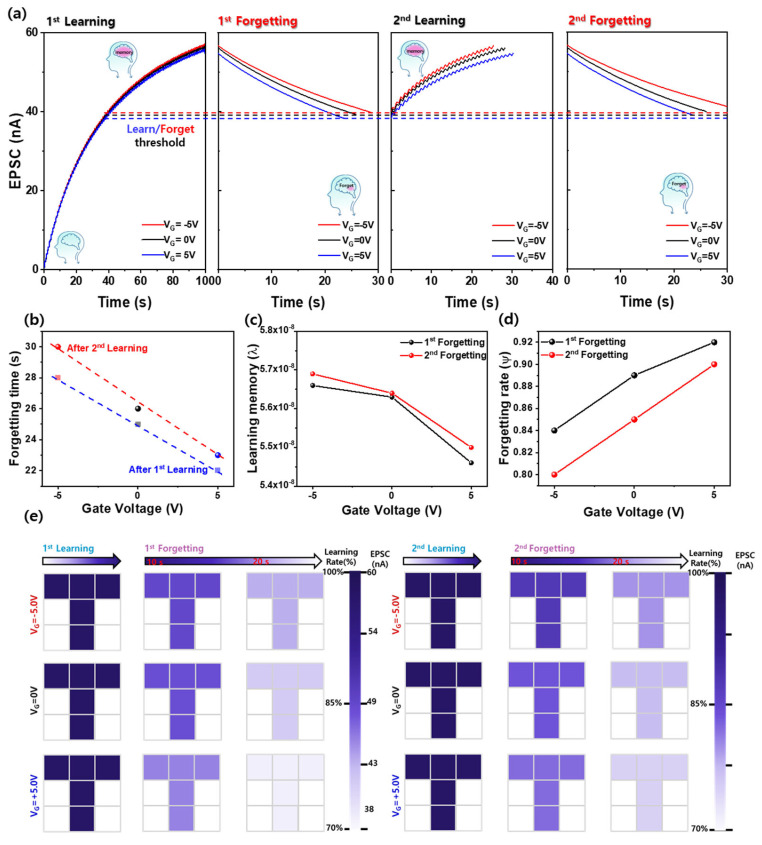
(**a**) Learning–forgetting behavior of the Al/ZnO NPs/SiO_2_/Si three-terminal optoelectronic synaptic device under UV stimulation (365 nm, 100 pulses, 0.5 s width, 50% duty cycle) and gate voltages of −5 V, 0 V, and +5 V. EPSC learning threshold defined at 70% of maximum EPSC. (**b**) Comparison of EPSC decay times (to 70% threshold) during the first and second forgetting processes. (**c**) Extracted long-term memory state (λ) and (**d**) forgetting rate (Ψ) based on Wickelgren’s power law fitting after the first and second forgetting cycles. (**e**) 3 × 3-pixel array visualization of in-sensing memory showing learning and forgetting dynamics through EPSC-based color encoding under varying gate voltages and repeated stimulation cycles.

Figure 7a presents the learning-forgetting experience behaviors of the ZnO NPs-based three-terminal optoelectronic synaptic devices under varying bottom gate voltages (V_G_ = −5.0 V, 0 V, +0.5 V). To simulate the learning process, a train of 365 nm UV pulses (100 cycles, pulse width: 0.5 s, duty cycle: 50%) was applied, resulting in gradual EPSC potentiation. Once the UV was turned off, the forgetting process was observed as an electrical depression of EPSC using different gate voltages (V_G_ = −5.0 V, 0 V, +0.5 V). A learning threshold was defined as a 70% level was used to evaluate forgetting [34]. The device exhibited gate-voltage-dependent modulation of both learning and forgetting behaviors. At a gate voltage of −5.0 V, the maximum EPSC reached 57 nA, with a corresponding learning threshold (70% maximum) of 39.8 nA. At 0V and +5.0 V, the maximum EPSCs were 56 nA and 54 nA, respectively, yielding thresholds of 38.7 nA and 37.6 nA. These results indicate that the EPSC amplitude of the ZnO NPs-based three-terminal device can be effectively tuned by gate bias, which modulates the carrier density in the ZnO channel through gate-induced electron injection or extraction via leakage through the SiO_2_ gate dielectric. After reaching the maximum EPSCs under each gate voltage condition, the first forgetting process is initiated by switching off UV illumination. The time required for EPSC to decay to the 70% threshold was 28 s (−5.0 V), 25 s (0 V), and 22 s (+5.0 V), indicating a slower decay rate under negative gate bias, as shown in Figure 7b. This trend suggests that the forgetting time is approximately proportional to the applied gate voltage, supporting the interpretation that the carrier dynamics are modulated by direct gate-assisted electron transfer rather than pure field effect. Following the initial forgetting process, a secondary learning process was carried out by reapplying UV pulses from the 70% EPSC level. The number of pulses required to recover the original maximum EPSC decreased to 25, 29, and 31 pulses for −5.0 V, 0 V, and +5.0 V, respectively. This reduction in the number of pulses compared to the first learning process improved learning efficiency due to memory reinforcement. Furthermore, the second forgetting time (time required for EPSC to decay to 70% of the maximum) increased to 34 s (−5.0 V), 26 s (0 V), and 23 s (+5.0 V), demonstrating a slower decay compared to the first cycle and reflecting enhanced memory retention upon repeated simulation. Figure 7b summarizes the forgetting times extracted from both cycles, revealing an approximately linear dependence on gate voltage. These findings confirm that EPSC retention and forgetting behavior can be finely modulated by gate-induced carrier control through gate leakage paths, even in the absence of strong electrostatic field effects. In particular, negative gate bias significantly improves retention by enhancing electron accumulation in the ZnO channel and promoting surface oxygen desorption, as illustrated in Figure 4h. To evaluate the long-term memory behavior and forgetting dynamics of the Al/ZnO NPs/SiO_2_/Si three-terminal synaptic device, Wickelgren’s power law, commonly used to describe biological forgetting, was employed [35]. The memory decay was fitted using the following Equation (1) [35]:

I = λ × (1 + β × t)^−Ψ^ (1)

where I represent the memory intensity (EPSC), t is the decay time, λ denotes the degree of learning or the long-term memory state at t = 0, β is the scaling parameter, and Ψ is the forgetting rate. Figure 7c shows the extracted long-term memory values (λ) after the first and second forgetting processes under different gate voltages. Repeated learning using the same pulse with the first learning process led to a slight increase in long-term memory across all conditions, indicating reinforcement of memory storage. Especially, long-term memory increased from 5.66 × 10^−8^ to 5.69 × 10^−8^ at −5.0 V, from 5.63 × 10^−8^ to 5.64 × 10^−8^ at 0 V, and from 5.46 × 10^−8^ to 5.50 × 10^−8^ at +5.0V. This enhancement is attributed to improved charge trapping and accumulation upon repeated stimulation, which strengthens synaptic weight and mimics long-term memory formation. Figure 7d presents the forgetting rate (Ψ) extracted from the fitted decay curves for the first and second forgetting cycles. The forgetting rate decreased after the second learning cycle at all gate voltages, with the most significant reduction observed under −5 V bias, confirming enhanced memory retention upon repeated learning. This effect is primarily attributed to the negative gate voltage, which increases electron concentration in the ZnO NP channel by injecting electrons through gate leakage across the SiO_2_ dielectric, thereby promoting oxygen desorption and suppressing carrier recombination. As a result, photogenerated carriers persist longer, delaying the EPSC decay and leading to reduced Ψ values. These results demonstrate that both learning repetition and gate-induced carrier modulation are effective strategies for tuning long-term synaptic plasticity in ZnO NP-based devices. Figure 7e illustrates a 3 × 3 visual memory array composed of nine Al/ZnO NPs/SiO_2_/Si optoelectronic synaptic devices, each subjected to different biases (V_G_ = −5.0 V, 0 V, +5.0 V). The EPSC values generated by 100 cycles of 365 nm UV pulse simulation (0.5 s pulse width, 50% duty cycle) were mapped as pixel intensities, representing the learning state of each device. Following the first learning cycle, the pixel color intensity (from dark to light blue) reflects the magnitude of EPSC, with stronger memory states corresponding to darker blue. As the UV was turned off, a time-dependent fading in pixel intensity was observed during the 1st forgetting process, with all pixels reaching the 70% EPSC threshold within 23 s, consistent with Figure 7a. 

In the second learning–forgetting cycle, the ZnO NPs-based 3-terminal optoelectronic synaptic devices were re-exposed to the same UV pulse conditions used during the first learning phase. Regardless of the gate voltage (−5 V, 0 V, +5 V), the devices successfully reached their respective maximum EPSC values, resulting in uniform encoding across all pixels with equally dark blue intensities, indicating full memory reactivation. During the second forgetting process, the decay of EPSC after UV-off was again visualized through pixel brightness. At 10 s after UV cessation, the device under −5 V still maintained a deep blue color, while those at 0 V and +5.0 V exhibited noticeably lighter shades, similar to the trend observed during the first forgetting. At 23 s—defined as the threshold for reaching 70% of the maximum EPSC—the −5.0 V-biased device continued to display strong color intensity, whereas the 0 V and +5 V-biased devices had faded considerably, approaching near-white levels. Compared to the first learning-forgetting process, however, all devices retained a stronger color tone at both 10 s and 23 s time points, suggesting a slower EPSC decay and improved memory retention after repeated stimulation. This enhancement in retention is consistent with the reduced forgetting rate observed in Figure 7d and is particularly prominent under negative gate bias, where carrier accumulation and oxygen desorption are more effective. Table 1 summarizes the synaptic performance of various reported optoelectronic synaptic devices and compares them with the results of our ZnO NPs-based three-terminal device. Key metrics such as excitatory post-synaptic current (EPSC), paired-pulse facilitation (PPF), and decay time (τ) are listed to evaluate short-term plasticity characteristics across different device structures. Our device exhibits a moderate EPSC of 60 nA, which is comparable to or higher than other oxide-based systems such as A-ZnO microtube/graphite (5 nA) and W/IGZO/HfAlOx/CeOx/W (4.5 nA) while maintaining much lower operational current than organic or Ta_2_O_5_-based systems that operate in the μA range [36,37,38,39,40,41]. Notably, the PPF ratio of 185% in our device is the highest among the reported devices, indicating superior short-term synaptic enhancement under paired-pulse input. The decay time of 26 s reflects strong retention of photogenerated carriers, which is beneficial for emulating biological forgetting behavior. This balance of high PPF, low power operation, and long decay time underscores the potential of our ZnO NPs-based device for low-power, light-modulated neuromorphic systems. Moreover, the use of a simple Al/ZnO/SiO_2_/Si architecture and back-gated control allows scalable integration with existing CMOS technology.

## 4. Conclusions

In this study, a three-terminal optoelectronic synaptic device based on Al/ZnO NPs/SiO_2_/Si structure was fabricated and characterized to emulate light-driven neuromorphic behavior. The device exhibited robust gate-tunable photoresponse under UV illumination, with enhanced photocurrent and memory retention under negative gate bias. Synaptic functions such as excitatory post-synaptic current (EPSC), paired-pulse facilitation (PPF), and short- and long-term memory behaviors were systematically evaluated under varying optical and electrical stimulation. The device demonstrated efficient optical potentiation and electrically modulated depression, with memory reinforcement evident from faster learning and slower forgetting in repeated cycles. Wickelgren’s power law analysis further confirmed the gate-dependent modulation of forgetting rate and long-term memory retention. A 3 × 3-pixel array simulation visualized visual memory formation and decay, highlighting spatial uniformity and improved retention under negative gate bias. These findings confirm that gate-induced carrier injection via SiO_2_ leakage in ZnO NPs enhances synaptic plasticity and memory characteristics, enabling controllable neuromorphic learning and forgetting. This work establishes ZnO NPs-based optoelectronic synaptic devices as promising candidates for in-sensor neuromorphic computing and artificial visual memory systems.

## Figures and Tables

**Table 1:** Comparison of reported optoelectronic synaptic devices with various material systems and configurations. Key synaptic performance parameters, including excitatory post-synaptic current (EPSC), paired-pulse facilitation (PPF), and decay time, are listed.

Modified Device Structure	Device Structure	Functionalities	EPSC (nA)	PPF (%)	Decay Time (s)	Refs.
2-terminal	A-ZnO Microtube/Graphite	EPSC, PPF, LTP	5 nA	180%	33s	[36]
Bottom gate	PDPP3T/SiO_2_/Si	EPSC, PPF	101 µA	71%	25s	[37]
Bottom gate	CrSbₓ/PtS_2_/Al_2_O_3_/Si	EPSC, PPF, LTM, STM	25 nA	150%	24s	[38]
Bottom gate	W/IGZO/HfAlOx/CeOx/W	EPSC, PPF, STP	4.5 µA	107%	12s	[39]
Top gate	TiN/HZO/NiSi_2_/Si	EPSC, PPF, LTP, STP	70 nA	80%	50µs	[40]
Bottom gate	ITO/Ta_2_O_5_/Si	EPSC, PPF	9 µA	150%	40ms	[41]
Bottom gate	Al/ZnO NPs/SiO_2_/P-Si	EPSC, PPF	60 nA	185%	26s	Our Work

## Data Availability

The data presented in this study are available on request from the corresponding author. The data are not publicly available due to privacy concerns.

## Supplementary Materials

The following supporting information can be downloaded at: https://www.mdpi.com/article/10.3390/nano15120908/s1.
