# Supplementary material for: Gate-Controlled Three-Terminal ZnO Nanoparticle Optoelectronic Synaptic Devices for In-Sensor Neuromorphic Memory Applications
Source: Nanomaterials (Basel). 2025 Jun 11;15(12):908. doi: 10.3390/nano15120908 (PMC12196014; doi:10.3390/nano15120908)
Supplement: Supplementary file 1 [file nanomaterials-15-00908-s001.zip › nanomaterials-3631950-supplementary.pdf]

Gate-Controlled Three-Terminal ZnO Nanoparticle Optoelectronic Synaptic Devices for In-Sensor Neuromorphic Memory Applications

Dabin Jeon[1], Seung Hun Lee[1], Sung-Nam Lee[1,2*]

[1]Department of IT Semiconductor Convergence Engineering, Tech University of Korea, Siheung 15073, Republic of Korea

[2]Department of Semiconductor Engineering, Tech University of Korea, Siheung 15073, Republic of Korea

Correspondence: snlee@tukorea.ac.kr; Tel.: +82-31-8041-0721

S1. I_{DS}–V_G characteristics of the Al/ZnO NPs/SiO_2/Si three-terminal device measured under dark and UV illumination

S1. I_{DS}–V_G characteristics of the Al/ZnO NPs/SiO_2/Si three-terminal device measured under dark and UV illumination

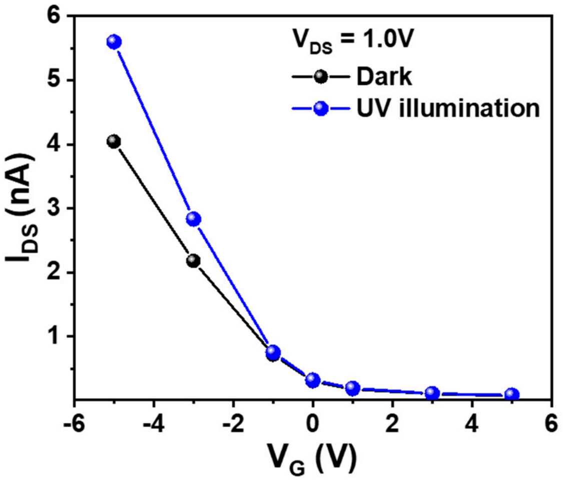

Figure S1. I_{DS}–V_G characteristics of the Al/ZnO NPs/SiO_2/Si three-terminal device measured under dark and UV illumination (λ = 365 nm) conditions at a fixed drain voltage of 1.0 V.

The device exhibits clear gate-tunable current modulation in both dark and UV-illuminated states. As the gate voltage increases from –5 V to +5 V, the drain current (I_{DS}) gradually decreases, suggesting that the modulation is governed by gate-voltage-dependent carrier injection through leakage current across the 100 nm SiO_2 dielectric. As shown in Figure 4h, when a negative gate voltage is applied, electrons are injected from the p-type Si back gate into the ZnO NP channel through the insulating SiO_2 layer, increasing the carrier density in the conduction path and thereby enhancing I_{DS}. In contrast, when a positive gate voltage is applied, electrons in the ZnO channel are drawn toward the gate and leak into the substrate, reducing the available carrier density in the conduction channel and resulting in a lower I_{DS}. This trend is more pronounced under UV illumination, where additional photogenerated carriers result in a higher current level across all gate voltages. At V_G = –5 V, I_{DS} reaches ~5.59 nA under UV and ~4.04 nA in the dark, while at V_G = +5 V, both currents reduce to ~86.6 pA and ~75.8 pA, respectively. The higher UV-induced current is attributed to the generation of electron–hole pairs and subsequent desorption of surface oxygen species, which release trapped electrons and enhance conductivity. These results confirm that the ZnO NP-based device supports synaptic behavior modulated by both optical input and gate-driven carrier injection via dielectric leakage, which is crucial for in-sensor neuromorphic applications.
